# Using meta-ethnography to understand the care transition experience of people with dementia and their caregivers

**DOI:** 10.1177/14713012211031779

**Published:** 2021-08-02

**Authors:** Marianne Saragosa, Lianne Jeffs, Karen Okrainec, Kerry Kuluski

**Affiliations:** Institute of Health Policy, Management and Evaluation, Dalla Lana School of Public Health, 206712 University of Toronto; 518775 Sinai Health, Canada; Institute of Health Policy, Management and Evaluation, Dalla Lana School of Public Health, 206712 University of Toronto; 518775 Sinai Health; Lunenfeld-Tanenbaum Research Institute, Canada; Institute of Health Policy, Management and Evaluation, Dalla Lana School of Public Health, 206712 University of Toronto; Open Lab, 7989 University Health Network; Division of General Internal Medicine, 7989 University Health Network; Department of Medicine, University of Toronto, Canada; Institute of Health Policy, Management and Evaluation, Dalla Lana School of Public Health, 206712 University of Toronto; Institute for Better Health, 5543 Trillium Health Partners, Canada

**Keywords:** dementia, health care transition, patient experience, caregiver experience, meta-ethnography

## Abstract

Older adults living with dementia are at risk for more complex health care transitions than individuals without this condition, non-impaired individuals. Poor quality care transitions have resulted in a growing body of qualitative empirical literature that to date has not been synthesized. We conducted a systematic literature review by applying a meta-ethnography approach to answer the following question: How do older adults with dementia and/or their caregivers experience and perceive healthcare transition: Screening resulted in a total of 18 studies that met inclusion criteria. Our analysis revealed the following three categories associated with the health care transition: (1) Feelings associated with the healthcare transition; (2) processes associated with the healthcare transition; and (3) evaluating the quality of care associated with the health care transition. Each category is represented by several themes that together illustrate an interconnected and layered experience. The health care transition, often triggered by caregivers reaching a “tipping point,” is manifested by a variety of feelings, while simultaneously caregivers report managing abrupt transition plans and maintaining vigilance over care being provided to their family member. Future practice and research opportunities should be more inclusive of persons with dementia and should establish ways of better supporting caregivers through needs assessments, addressing feelings of grief, ongoing communication with the care team, and integrating more personalized knowledge at points of transition.

## Introduction

Concerns of poor quality health care transitions among persons with complex care needs are well documented in the literature (Cheek et al., 2006; Coleman, 2003). Existing evidence suggests that *health care transitions*, defined as patient movement between and within health care settings and from different health professionals for the purpose of receiving care (World Health Organization, 2016), are points of vulnerability that contribute to higher rates of adverse outcomes (Arora et al., 2010; Mudge et al., 2013), unnecessary health care use (Gruneir et al., 2012; Teno et al., 2018) and health care spending (Comans et al., 2016). Patients older than 65 years with cognitive and functional impairment are particularly at risk for poor transitions (Gruneir et al., 2012; Mondor et al., 2017).

There is a well-established association between multimorbidity (chronic disease burden) and dementia and health care utilization (Mondor et al., 2017; Public Health Agency of Canada, 2014). Dementia (including Alzheimer disease) is a progressive degenerative condition that includes memory loss, language deficits, and behavioral problems (Alzheimer’s Association, 2019). Individuals who develop dementia often require more care and accordingly have more health care transitions when compared to older adults without this condition (Callahan et al., 2015a, 2015b). Health care transitions have been identified as an issue for people living with dementia in research and practice for many years and have become an area of focus in national strategies and guidelines (Public Health Agency of Canada, 2019; Scottish Government, 2017). Frequent transitions of locations can interrupt continuity of care, impact negatively on patient experiences, contribute to ineffective communication, and increase delirium and falls (Chenoweth et al., 2015; Goldberg et al., 2015).

*Transitional care* is defined as a broad range of time limited services that have traditionally been designed to address better care coordination for patients at high risk of unplanned readmissions and their caregivers (Naylor et al., 2011). However, most transitional care programs fail to include or limit the inclusion of persons with dementia, despite evidence that this group has difficulty adjusting to new care environments (Sury et al., 2013). This failure appears to be because of exclusion criteria or unexplained or unacknowledged exclusion practices (e.g., The Care Transition Intervention) (Hirschman & Hodgson, 2018; Piraino et al, 2012; Taylor, et al., 2012). Instead, pre-existing interventions on transitions in care have been adapted for persons living with dementia, or interventions, for example, meant to support the transition from home care to residential living target only family caregivers of people with dementia (Hirschman & Hodgson, 2018; Mueller et al., 2017). Yet, engaging persons with dementia and their caregivers in the research process is an increasingly common approach to improving value and relevance of research outcomes (Bethell et al., 2018). Going forward, improving health care transitions for persons with dementia and their families requires a collective understanding of both the individual and dyadic experiences of health care transitions.

A common way of combining knowledge available from primary qualitative studies is through synthesis. It is argued that this approach can produce novel interpretation of findings that is more substantive than those resulting from individual primary studies (Finfgeld, 2003). To our knowledge, there has not been a meta-ethnography of the care transition experience of persons with dementia and their caregivers. By exploring patients with dementia and their caregivers’ perception of health care transitions, we attempt to start the groundwork for a new theoretical interpretation that could be more accessible to clinicians, researchers, and policy makers (Finfgeld, 2003) and potentially shed light on where to focus service improvements. Severity of disability and dependency among those living with Alzheimer’s disease and other dementias is having a significant and growing impact globally (Public Health Agency of Canada, 2019). With the global prevalence of Alzheimer disease and related dementias estimated to be 50 million in 2019, and expected to rise to 65.7 million by 2030, identifying gaps in the quality of transitional care for people with dementia is exceedingly critical (Prince et al., 2013; World Health Organization, 2019).

### Aim and research question

The aim of this meta-ethnography was to systematically search and present a synthesis of the qualitative health care transition literature involving people with dementia or cognitive impairment, and/or caregivers or relatives of persons living with dementia. The research question was *How do older adults with dementia and/or their caregivers experience and perceive health care transitions?*

## Methods

### Meta-ethnography overview

A meta-ethnographic approach was used to systematically compare a set of studies that pertained to the research question (Noblit & Hare, 1988). In contrast to other narrative-type literature review methods, meta-ethnography attempts to achieve a new interpretation of the selected studies, moving toward a reconceptualization (Britten et al., 2002; Doyle, 2003), or to generate theory by synthesizing qualitative evidence (France et al., 2019). The product of this synthesis is the translation of studies into one another, which requires the researcher to capture trends across qualitative studies in the form of common ideas, concepts, and metaphors to produce a novel interpretation of a phenomenon that transcends individual study findings (Britten et al., 2002).

### Literature search

Consultation with an academic librarian facilitated the development of a search strategy. Five databases were searched (CINAHL, Embase, PsycINFO, Medline, and AgeLine) from 2009 to 2019 using a combination of medical subject headings and free text terms (Supplemental Appendix 1 for full list). Relevant terms such as “care transition,” “care in transition,” “dementia,” and “cognitive disorder” were used along with spelling variations to complete the search.

### Selection criteria

The study focuses on qualitative studies of the care transition experiences across the health care system of persons 65 years or older living with dementia or cognitive impairment and/or their family and friend caregivers. Studies were accepted if they used mixed or multi-methods; however, only the qualitative findings were considered. Only papers written in English were included, and all types of care transitions—between care settings and within—were considered as our interest lay in understanding a range of transitions. Finally, studies had to be peer-reviewed and have ethics approval. Studies were excluded if they only included the views or experiences of health care professionals or lacked a focus on experiences during health care transitions.

### Data analysis

The first author extracted the following data from included studies to describe study characteristics: author; year of publication; study country of origin; principal experiences explored (i.e., study aim(s)); care transition type; methodology; data collection method(s); and number and type of study participants (patients with dementia and/or their caregivers). Next, to determine how the studies were related, a table was created to display concepts and themes across all studies (Atkins et al., 2008). Authors extracted verbatim the studies’ themes and associated direct quotes listed as second- and first-order constructs, respectively. Definitions of first-, second-, and third-order constructs have been provided in Table 1 (Purc‐Stephenson & Thrasher, 2010). Study authors used a constructivist philosophical position informed by their past and present professional experiences and research practices in the analytical process (Charmaz, 2014).

**Table 1.** Definition of first-, second-, and third-level constructs.

Term	Definition
First-order constructs	The participant’s interpretation of their experience with caregiving.
Second-order constructs	The author’s interpretation of participants’ experience with health care transitions, which are described as themes and concepts using the original terminology presented in the papers.
Third-order constructs	The synthesis team’s interpretation of how the studies relate by comparing first- and second-order constructs and conveying overarching themes and concepts about a phenomenon.

Given the relative heterogeneity of themes across the studies, study authors conducted a thematic analysis by open coding the extracted direct quotes (referring to the original papers for context as necessary) to identify several categories. These categories included, for example, “Ad hoc discharge planning,” “communication gaps,” and “being ignored.” Following closely Atkins et al. (2008), we then continued to compare the studies, which allowed for the initial categories to be refined and merged. Through this translation authors identified a higher interpretation of three major categories and their themes. In our case, this step was done by the first author and then considered, discussed, and agreed upon with all authors.

## Findings

### Selection of studies

**Figure 1.**
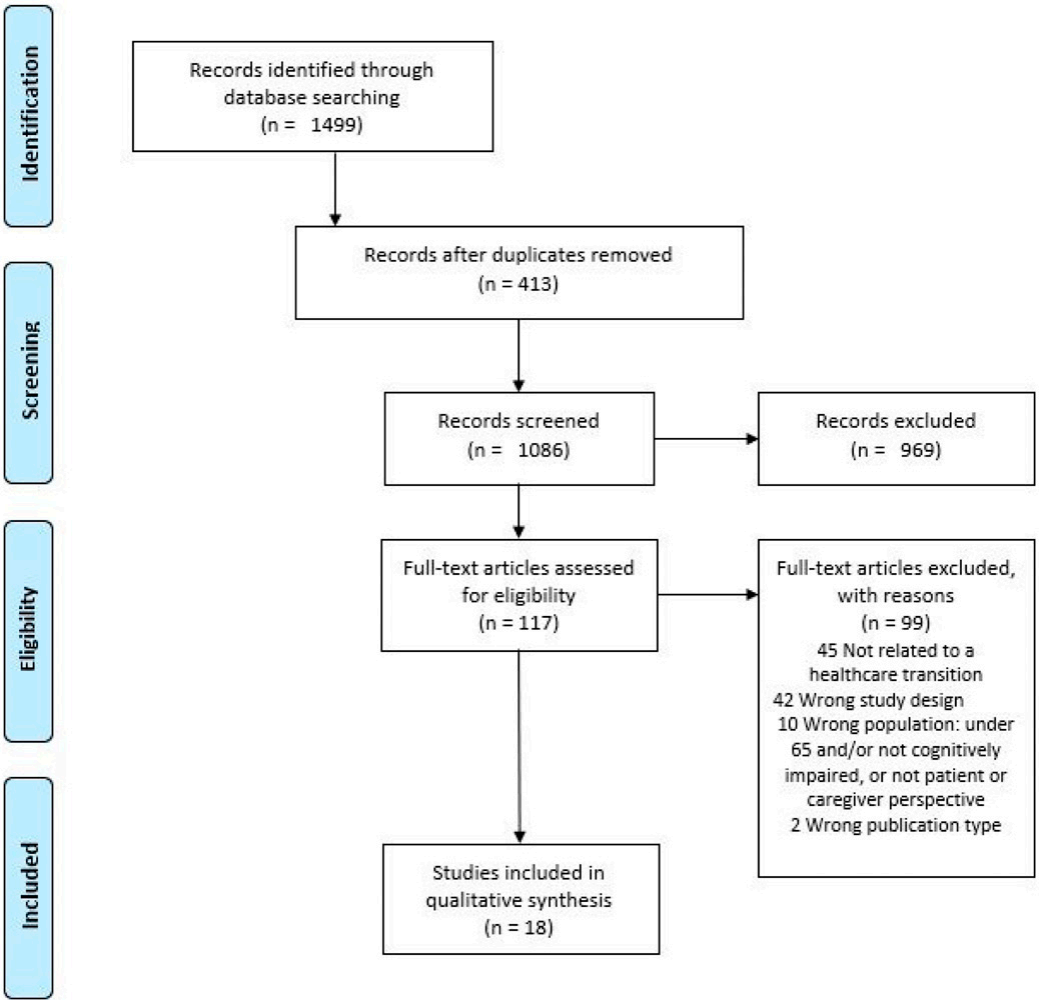
Selection of studies.

Our electronic database search yielded a total of 1499 studies, of which 413 duplicates were removed. The selection process of the remaining studies (*n* = 1086) involved three phases. An initial title screen followed by an abstract review was performed by the first author (see Box 1 for inclusion and exclusion criteria). The final screening stage involved evaluating full text papers against inclusion criteria leaving 18 studies. Reasons for excluding full text articles included studies not focused on a transition in care (*n* = 45); wrong study design (*n* = 44); and wrong study population (*n* = 10). Figure 1 illustrates the PRISMA flow diagram of the selection process.

**Box 1. Search criteria.**

**Table:** 

Inclusion criteria
•Empirical studies published in English from 2009 onward.
•Studies targeting older adults living with dementia or cognitive impairment, or family and friend caregivers of persons living with dementia or cognitive impairment.
•Applied qualitative methods, including mixed or multi-methods studies.
•Reported on the experience of health care transitions either by older adults with dementia or by family caregivers or both.
Exclusion criteria
•Studies that reported on the experience and perceptions of health care transitions by only health care professionals.
•Participants diagnosed with dementia younger than 65 years.
•Studies that employed only quantitative methods.
•Published in non–peer-reviewed journals, gray literature, commentaries, or conference abstracts.

### Study quality

Study authors used the 32-item checklist consolidated criteria for reporting qualitative research (COREQ) to assess quality of reported information in included studies (Tong et al., 2007). The number of items reported varied (see Table 2). Most studies reported on sample size, sampling, data and consistent findings, quotations present, and clarity of major themes. Conversely, items least reported included personal characteristics of the researchers, relationship status between researcher and participants, and data saturation. None of the studies mentioned transcripts being returned to study participants. Despite reporting variation, no studies were excluded based on the COREQ assessment as evidence does not support this will improve the quality of this review (Dixon-Woods et al., 2007); rather, the assessment was used to support recommendations for future dementia research.

**Table 2.** Quality assessment according to the consolidated criteria for reporting qualitative research.

Criteria	References of studies reporting each criterion																	
	Bauer et al., (2011a)	Bauer et al., (2011b)	Bloomer et al. (2016)	Bramble et al. (2009)	Busean and Twigg (2014)	Cloutier and Penning (2017)	Crawford et al. (2015)	Digby et al. (2012)	Fitzgerald et al. (2011)	Henkusens et al. (2014)	Hutchings et al. (2011)	Jamieson et al. (2016)	Kelsey et al. (2010)	Kiwi et al. (2018)	McLennon et al. (2019)	Mockford et al. (2017)	Parke et al. (2013)	Wladkowski (2016)
Domain 1: Personal characteristics																		
Personal characteristics																		
1. Interviewer or facilitator				●				●										
2. Credentials				●		●		●	●				●		●	●		●
3. Occupation		●		●		●	●	●	●			●				●		
4. Gender						●						●						
5. Experience and training							●				●	●						
Relationship with participants																		
6. Relationship established			●	●		●	●	●		●			●					
7. Participant knowledge of the interviewer				●			●											
8. Interviewer characteristics						●	●											
Domain 2: Study design																		
Theoretical framework																		
9. Methodological orientation and theory	●		●	●		●	●	●	●	●	●						●	
Participant’s selection																		
10. Sampling	●	●	●	●	●		●	●	●	●	●	●	●	●	●	●	●	●
11. Method of approach	●	●	●	●			●	●			●	●	●			●	●	●
12. Sample size	●	●	●	●	●	●	●	●	●	●	●	●	●	●	●	●	●	●
13. Non-participation							●	●								●	●	
Setting																		
14. Setting of data collection	●		●	●			●	●		●		●	●				●	●
15. Presence of non-participants							●											
16. Description of sample	●	●	●	●		●	●	●	●	●	●	●	●				●	●
Data collection																		
17. Interview guide			●				●	●	●				●	●			●	
18. Repeat interviews										●						●		
19. Audio or audiovisual recording	●		●	●			●	●	●		●	●	●	●		●		
20. Field notes					●			●			●							
21. Duration	●		●	●			●			●	●			●			●	
22. Data saturation									●	●	●						●	
23. Transcripts returned to participants																		
Domain 3: Analysis and findings																		
Data analysis																		
24. Number of data coders				●			●				●		●	●	●		●	
25. Description of the coding tree				●			●		●		●	●	●	●		●		
26. Derivation of themes	●	●	●	●			●	●	●	●	●	●	●	●	●	●		●
27. Software	●				●				●				●			●	●	●
28. Participant checking	●								●			●				●		●
Reporting																		
29. Quotations presented	●	●	●	●	●		●	●	●	●	●	●	●	●	●	●	●	●
30. Data and findings consistent	●	●	●	●	●	●	●	●	●	●	●	●	●	●	●	●	●	●
31. Clarity of major themes	●	●	●	●		●	●	●	●	●	●	●	●	●	●	●	●	●
32. Clarity of minor themes/ or diverse cases			●	●		●	●	●	●	●	●	●	●	●				

### Study characteristics

Table 3 includes the key characteristics of the 18 included papers that represented 16 studies. Country of publication included Australia (*n* = 8), followed by Canada (*n* = 4), the USA (*n* = 3), the UK (*n* = 2), and Sweden (*n* = 1). Most of the studies considered the experience associated with the home or hospital to long-term care transition trajectory (*n* = 9). A key feature across most studies was the reliance on the caregiver perspective whereby only five studies reported on the experience of the person with dementia; one study interviewed recently admitted residents with dementia (Digby et al., 2012) and four involved patient–caregiver dyads (Buse & Twigg, 2014; Henkusens, et al., 2014; Mockford et al., 2017; Parke et al., 2013). Collectively the studies captured the perspectives of 244 caregivers compared to 49 persons living with dementia. Only three of these studies included the direct quotes of those with dementia (Buse & Twigg, 2014; Digby et al., 2012; Henkusens et al., 2014).

**Table 3.** Characteristics of the included studies.

Author/s and year	Journal	Country	Aim of study	Type of participants (no.)	Data collection	Methodology
Bauer et al., (2011a)	Journal of Healthcare Quality	Australia	Perceptions of family carers of older people with dementia who had made the transition through the hospital system	Caregivers (25)	Interviews	Qualitative constructivist
Bauer et al., (2011b)	Dementia	Australia	To report on the experiences of family carers of hospital discharge planning process for their family members with a dementia	Same as Bauer et al. 2011	Interviews	Qualitative research
Bloomer et al. (2016)	Dementia	Australia	To explore the carer’s experience when the person he/she cares for is an inpatient in hospital.	Caregivers (20)	Interviews	Descriptive qualitative
Bramble et al. (2009)	Journal of Clinical Nursing	Australia	To provide in-depth descriptions of the experiences of family caregivers when placing their relative with dementia in LTC	Caregivers (10)	Interviews	Qualitative descriptive
Buse and Twigg (2014)	Journal of Aging Studies	United Kingdom	To analyze the role of handbags in the everyday lives of women with dementia	Patients (13) Caregivers (29)	Observations, interviews, and visual and sensory approaches	Ethnographic
Cloutier and Penning (2017)	The Gerontologist	Canada	To identify lessons that our mothers’ experiences offer for the care of older women with dementia	Caregivers (2)	Personal narratives	Life-course perspective
Crawford et al. (2015)	Aging & Mental Health	Australia	To provide in-depth descriptions of the experiences of caregivers during their transition from day-to-day caregiver of a person with dementia to a visitor in an LTC facility	Caregivers (20)	Interviews	Qualitative descriptive
Digby et al. (2012)	International Journal of Older People Nursing	Australia	To understand how older patients with mild to moderate dementia experienced the transfer from acute to subacute care and settling-in period	Patients (8)	Interviews	Qualitative descriptive
Bauer et al., (2011b)	Australian Health Review	Australia	To understand the family carers’ experience of hospital discharge planning; understand how well the discharge plan for patients with a dementia met the needs of the family carer; and ascertain what improvements family carers thought could better assist the transition from hospital to residential, subacute, or home-based care	Same as Bauer et al. 2011	Interviews	Qualitative constructivist
Henkusens et al. (2014)	Journal of Applied Gerontology	Canada	To examine post relocation (a) the experience of mealtimes and how they differ from meals at home; (b) how the LTC home environment contributes to this experience; and (c) the strategies that PWD and their family CP use to respond to these new mealtime experiences that resulted from the relocation to LTC	Patients (7) Caregivers (7)	Interviews	Longitudinal grounded theory
Hutchings et al. (2011)	Canadian Journal on Aging	Canada	To provide insight into the experience of family members with the relocation of residents with dementia from an institutional environment to an assisted living environment	Caregivers (10)	Interviews	Hermeneutic phenomenology
Jamieson et al. (2016)	Dementia	Australia	To describe the experience of carers when the person with dementia transitions home from hospital	Caregivers (30)	Interviews	Qualitative design
Kelsey et al (2010)	American Journal of Alzheimer’s Disease & Other Dementias	USA	To describe the decision to move a family member with Alzheimer’s disease and related dementias from the community to an assisted living facility and from there to a memory unit, from the perspective of the primary caregiver	Caregivers (15)	Interviews	Grounded theory
Kiwi et al. (2018)	Journal of Cross-Cultural Gerontology	Sweden	To analyze and understand how relatives reason and argue around making decisions about moving family members with dementia to nursing homes	Caregivers (20)	Interviews	Not reported
McLennon et al. (2019)	Clinical Nursing Research	USA	To identify needs, concerns, and advice from the blogs of caregivers caring for a person with dementia at the end of life	Caregivers (6)	Blogs	Qualitative descriptive
Mockford et al. (2017)	Health Expectations	UK	To develop service user-led recommendations to enable smooth transition for people living with memory loss from acute hospital to the community which will be disseminated to health and social care professionals involved in hospital discharge planning	Patients (15) Caregivers (15)	Interviews	Framework analysis
Parke et al. (2013)	International Journal of Nursing Studies	Canada	To identify factors that facilitate or impede safe transitional care in the ED for community dwelling older adults with dementias	Patients (6) Caregivers (11)	Interviews, photographic narrative journal, photo elicitation	Interpretive, descriptive exploratory
Wladkowski (2016)	Journal of Social Work in End-of-Life & Palliative Care	USA	To explore the impact of live discharge on the caregiver of individuals with a dementia diagnosis who lost hospice services due to prognosis improvement or failure to maintain ongoing eligibility	Caregivers (24)	Interviews	Not reported

### Synthesis findings

Our study findings were organized into three major categories and a total of eight themes that reflected the layered experiences of caregivers and persons with dementia either before, during, or after the health care transitions: (1) Feelings associated with the health care transition; (2) Processes associated with the health care transition; and (3) Evaluating the quality of care associated with health care transition. The themes are italicized under each of the categories. Illustrative first- and second-order constructs are outlined in Table 4.

**Table 4.** Themes derived from included studies.

Category	Second-order constructs (interpretations of primary study authors)	First-order construct (illustrative quotations)
Feelings associated with the health care transition		
Facing an increase in challenging behaviors—reaching a “tipping point”	Caregivers mentioned that the hospital admission was the final tipping point for them; they had come to the realization that they could no longer care for their relative or friend at home and residential care was the only option (Crawford, et al. 2015)	Caregiver: “I wasn’t getting a lot of sleep. If I didn’t wake up when he was starting to get out of bed, he’d get up and then he’d have a fall. He wouldn’t wait for me to put the light on.” (Crawford et al., 2015, p. 741)
	Health care professionals incorrectly assumed caregivers’ capacity to continue the role that he/she has been playing previously (Bloomer, et al. 2016)	Caregiver: “. . . Well, when they were talking about sending him home, I had the horrors because there’s no way I could do any more than what I was doing” (Bloomer et al., 2016), p. 1241
	Lack of physical strength contributed to caregivers experiencing that taking care of the person with dementia, the rest of the family and themselves was too much to handle (Kiwi et al. 2018)	Caregiver: “I understood taking care of mom all of these years was not an easy job at all. Because I have had a herniated disc in my neck and couldn’t help her. I got help from the municipality for cleaning mom’s apartment, I noticed that my hands would go numb, finally, I became ill. I could not help myself or mother any longer” (Kiwi et al., 2018, p. 30)
Feeling ignored and undervalued	In high stress environments, like the Emergency Department, older adults and caregivers felt ignored, forgotten, and unimportant (Parke et al., 2013)	Caregiver: “and he [physician] just walked by and just took off and never talked to me at all, knowing that I was waiting for the results of those tests” (Parke et al., 2013, p. 1214)
	Many caregivers perceived health care providers undervalued family caregivers as a resource (Fitzgerald et al., 2011)	Caregiver: “I told the aged care doctor ‘I don’t think you should be sending my husband home’. When they did send him home… the doctor rang me up on the Saturday morning and said, ‘Vera, get your husband back here. He’s got blood poisoning. . .’” (Fitzgerald et al., 2011, p. 368)
Feelings of grief and loss of purpose	The health care transition tended to trigger loss, sadness, and relentless grief among caregivers, worsened by the lack of preparation for the transition (Bramble, et al., 2009)	Caregiver: “…it’s guilt, it’s grief, sadness, regret, all that and you don’t think this will happen” (Bramble et al., 2009, p. 3121)
Being ready for the care transition—Starting to live a new life	Feelings of relief and liberation was experienced by caregiver’s post placement (Kiwi et al., 2018)	Caregiver: “I have personally decided to give myself a chance to live a life with my children. I hope that my new life after my mother has moved to a nursing home will be a normal life without complications” (Kiwi et al., 2018, p. 36)
Processes associated with the health care transition		
Perceiving “ad hoc” discharge planning	Family caregivers perceived discharge planning to be ad hoc and in some cases a discharge plan was not apparent (Bauer et al., 2011b)	Caregiver: “We only ever found out [about discharge] because my brother was there one day and one of the nurses said, ‘Oh, she’ll probably be going home tomorrow’” (Bauer et al., 2011a)
Gaping holes in care coordination, continuity of care, and perceived support	Gaps in uncoordinated discharge and delays in (re)starting community care services often exacerbated caregiver anxiety (Jamieson et al., 2016)	Caregiver: “He needed more care when he got home, I had to make an ACAT [Aged Care Assessment Team] assessment appointment, phone social workers, lots of phoning but no care, it took several weeks, then when they actually assessed him they said he didn’t qualify for more care. In the end I got a private carer” (Jamieson et al., 2016, p. 1117)
Evaluating the quality of care associated with the health care transition		
Questioning quality of care	Many caregivers expressed concern health care providers ability to care for persons living with dementia (Bauer et al., 2011a)	Caregiver: “When she went in, she was sort of functionally continent. If you remind her and take her to the toilet; she’ll go to the toilet. Well that wasn’t happening . . . They were quite happy for her to sit in a pad and change it when it’s all wet. They didn’t understand the personal needs” (Bauer et al., 2011a, p. 319)
	Being treated patronizing by some staff members was noted by persons with dementia (Digby et al., 2012)	Persons with dementia: “…everyone’s darling, sweetheart, lovey and all the rest of it… and 1 day a big woman, a very big woman she called me ‘sweetheart’ and I certainly didn’t see myself in that role at all. I didn’t say it to her, but I thought to myself – huh!” (Digby et al., 2012, p. 61)
Being satisfied with quality of care	Persons with dementia who had transition to long-term care described positive experiences of “Holding on to Home” (Henkusens et al., 2014)	Persons with dementia: “And that’s probably part of why I enjoy the food so much, is that I’m not eating by myself now” (Henkusens et al., 2014, p 551)
	Satisfied with care provided in the caring staff and meaningful activities (Kelsey et al., 2010)	Caregiver: ‘‘I’d say they even surpassed my expectations. I thought they did a wonderful job . . . one special nurse that took care of him was such a loving, caring young woman. She was wonderful.’’ (Kelsey et al., 2010, p. 260)

#### Feelings associated with the health care transition

##### Facing an increase in challenging behaviors—reaching a “tipping point.”

Noticeable changes in the person with dementia included memory and cognitive deterioration, sleeplessness, paranoia, and wandering behavior requiring constant supervision (Bloomer et al., 2016; Bramble et al., 2009; Crawford et al., 2015; Kelsey et al. 2010; Kiwi et al. 2018). Four studies reported that challenging behavior led many to reach the “tipping point,” manifested in caregivers realizing that they no longer could care for the individual (Bauer et al., 2011a; Bramble et al., 2009; Crawford et al., 2015; Kiwi et al., 2018). For caregivers in three studies, caregiving was at the expense of their own declining physical and mental health (Bramble et al., 2009; Jamieson et al. 2016; Kiwi et al., 2018) as reported by one daughter feeling depressed and unable to cope with the added burden of her father during the “Pre-Decision” stage of the transition process,

I myself became depressed after losing my mother and now my father is as ill. I’m tired and cannot cope with extra problems. In the beginning my father began to behave differently and became more and more forgetful, I become his home care assistant, personal assistant, although, I myself was neither physically nor mentally a healthy person, but I tried to help him. (Kiwi et al., 2018, p. 29)

Feeling like they could no longer manage at home, caregivers expressed either themselves wanting to make this fact known or hoping that a health care professional would make the assertion that the person with dementia needed more care (Crawford et al., 2015; Kelsey et al., 2010). For some caregivers, they received professional medical feedback that the individual required more care to address risky behavior such as an environment that catered to wandering and exit-seeking behavior:

They talked to me about it, but I ultimately decided to move her mainly for her own safety, because she was getting dressed in the middle of the night and thinking she had to go to work . . . and trying to get out of the building. So it was for her own safety, but it was at the initiation mostly from the staff (Kelsey et al., 2010, p. 259).

Sometimes caregivers who experienced caregiving after a hospice to home transition questioned their ability to continue to provide care for an unknown amount of time. The live hospice discharge was viewed both as a gift of more time and as an opportunity to reassess the caregiving role, “I don’t want my mother to pass away, but can I do this for 3 more years? Can I do this for 2 more years?” (Wladkowski, 2016, p. 57).

##### Feeling ignored and undervalued

In five studies, caregivers reported that their contributions went unnoticed (Bauer et al., 2011a; Bloomer et al., 2016; Cloutier & Penning, 2017; Jamieson et al., 2016; Parke et al., 2013). For one husband, he perceived staff felt “annoyed” by his involvement, which may have led to a difficult relationship with health care providers,

I was as much help to them [the hospital staff] as anything, because I was doing quite a lot of the caring. I really did feel like I was annoying the staff and I don’t like feeling like that. I just had that impression. I would have liked someone to come and say, ‘Look, let’s sit down and I’ll explain it to you.’ Maybe, they’re just too busy, but it would have been helpful to me (Bauer et al., 2011a, p. 320).

However, sometimes health care providers did express appreciation to caregivers who continued to provide care during the hospitalization as mentioned by a wife,

I ducked home to change my clothes and I got a phone call. He was very restless and they were having trouble keeping him in bed. The next day they did thank me very much for helping them out overnight (Jamieson et al., 2016, p. 1116).

Caregivers expressed frustrations being ignored over their attempts to share personal information about the individual (Bramble et al., 2009). When helpful information was relayed to the clinical staff, it appeared that clinicians did not put the information into practice. As Bauer et al. (2011a) pointed out, if health care providers used the personalized knowledge about the person with a dementia, better individualized care could have been delivered and the transition would be better informed. This lack of communication or information sharing also led to persons with dementia and their caregiver feeling ignored: “…and he [physician] just walked by and just took off and never talked to me at all, knowing that I was waiting for the results of those tests” (Parke et al., 2013, p. 1214). Thus, feeling undervalued is heightened by caregivers’ close and intimate knowledge of the individual with dementia and wanting to protect their loved one.

##### Feelings of grief and loss of purpose

Seven of the papers contributed to the concept of the emotional toll of the transition experienced by persons with dementia and their family caregivers (Bloomer et al., 2016; Bramble et al., 2009; Cloutier & Penning, 2017; Crawford et al., 2015; Digby et al., 2012; Kelsey et al., 2010; Wladkowski, 2016). Particularly, caregivers described feeling a sense of “loss” usually in the context of being home alone and “having nothing to do” (Bloomer et al., 2016; Bramble et al., 2009; Crawford et al., 2015; Wladkowski, 2016). In some cases, spousal caregivers expressed strong emotional responses to the transition, including “relentless grief” rather than relief, and for some, they sought professional counseling noted in the following quote:

… I actually had professional help outside … I just don’t seem to be able to do enough. Can’t come enough, can’t say enough, can’t give enough. And the most difficult thing to deal with is knowing the conversations that won’t be had because the mind is going (Bramble et al., 2009, p. 3121).

Persons with dementia commented on feeling a “loss of control” in the context of being left out of the decision-making process about their care (Digby et al., 2012). One person decided to resist against staff intervention in the new residential environment, “I let them go ahead and do it, and then when they go out the door, I do it my own way. I’m not going to take it from anyone!” (Digby et al., 2012, p. 61).

##### Being ready for the care transition: Starting to live a new life

In response to the care transition, a subset of caregivers experienced a sense of relief knowing their family member was being taken care of in a safe environment (Crawford et al., 2015; Digby et al., 2012; Hutchings et al., 2011; Kelsey et al., 2010; Kiwi et al., 2018; Wladkowski, 2016). Crawford et al. (2015) talked about caregivers having to get used to a new life, and as one wife admitted, “You’ve got to be able to do things… I know he’s being well looked after. Look, I switch off from it as best I can because there’s no good me putting myself into bad health.” Participants in the study by Kiwi et al. (2018) who experienced ill health as a result of their caregiving responsibilities, reported: the nursing home “saved my life” and “we have the freedom to feel at home in our own home,” and “If I had wings, I could fly with joy…Now I can sleep quite easily.”

In one study which included interviews with people with dementia who relocated to a nursing home, participants expressed wanting to look forward rather than backward during the relocation, “The place being what it is, it might be terrible for me, but I think I’ll try and go back and see how I can manage” (Digby et al., 2012, p. 62). Thus, it was as important for persons with dementia to settle into a new environment as much as was for their family members (Hutchings et al., 2011; Kelsey et al., 2010). For example, caregivers also acknowledged relief and contentment when their family member transitioned to different supportive living settings (i.e., an assisted living and memory care unit ) (Hutchings et al., 2011; Kelsey et al., 2010). A relocation to an assisted living program was perceived to enhance a home-life environment and residents appeared happier.

#### Processes associated with the health care transition

##### Perceiving “ad hoc” discharge planning

Study authors Bauer et al. (2011b) used the term “ad hoc” to describe family caregivers lack of awareness of discharge planning. Caregiver participants were not always made aware of discharge plans, including discussions leading up to abrupt health care transitions of the patient with dementia. Becoming aware of the discharge seemed almost by coincidence. Caregivers in a study by Bauer et al. (2011b) noted that “discharge information came about randomly.”

Some family caregivers perceived discharge plans to be entirely decided by health care providers (Bauer et al., 2011a; Bloomer et al., 2016; Fitzgerald et al., 2011). In these cases, not consulting with caregivers resulted in caregivers missing crucial conversations or patients being discharged prematurely from the hospital. In the example below, a wife recounts,

I told the aged care doctor ‘I don’t think you should be sending my husband home’. When they did send him home, they sent him home far too early. And I nursed him through the night, all night. I got his temperature down but the doctor rang me up on the Saturday morning and said, ‘Vera, get your husband back here. He’s got blood poisoning. . .’ (Fitzgerald et al., 2011, p. 368).

In five studies, caregivers acted as advocates for the person with dementia during the discharge process (Bauer et al., 2011a, 2011b; Buse & Twigg, 2014; Fitzgerald et al., 2011; Jamieson et al., 2016; McLennon et al., 2019). At times, caregivers had to “assert” themselves to get quality care transition processes in place. One caregiver who was a granddaughter of the care recipient had to advocate for her grandmother upon entering a nursing home to be able to retain money in her bag:

…she has some cash on her, which the care home don’t like, but we have said to them ‘if she doesn’t have any money on her you’ll never hear the end of it’. You know, it’s like a security blanket for her (Buse & Twigg, 2014, p. 10).

##### Gaping holes in communication and support

Lack of communication or information sharing manifested in several ways. Caregiver participants in 10 studies reported a lack of a centralized person coordinating the transition. Instead, caregivers had to either seek out information from different providers or assumed they would be contacted, while others were left with little to no information (Bauer et al., 2011a, 2011b; Bloomer et al., 2016; Cloutier & Penning, 2017; Crawford et al., 2015; Fitzgerald et al., 2011; Kelsey et al., 2010; McLennon et al., 2019; Mockford et al., 2017; Parke et al., 2013). Mockford et al. (2017) illustrate this point with the following quote from a caregiver,

But I think it’s the sort of situation that if you’re a carer like myself, you could get very aggravated by it because there are so many people with their fingers in the pie…you get onto one person and then you have to get onto another (Mockford et al., 2017, p. 501).

Caregivers were not always told about changes in medication (Bauer et al., 2011a; Bloomer et al., 2016; Crawford et al., 2015) and perceived communication to be fragmented. In their views, this resulted in substandard care coordination post transition (Bauer et al., 2011a; Bramble et al., 2009; Fitzgerald et al., 2011; Jamieson et al., 2016; Kiwi et al., 2018).

Four studies illuminated frustrations with system-level barriers that impacted quality care coordination, including “breakdown in lines of communication” (Bauer et al., 2011a) and service boundaries governed by particular geographical areas (Jamieson et al., 2016; Kiwi et al., 2018). These rules not only complicated the health care transition but also placed undue burden back onto the caregivers who had to “navigate entry into services” (Jamieson et al., 2016).

Health care providers or formal and informal networks acted as important sources of information and support for caregivers in four studies. For some caregivers, this meant establishing a relationship and interacting with a member of the health care team, like a “social worker” (Crawford et al., 2015), and “physios and the occupational therapists” (Fitzgerald et al., 2011). Others spoke openly about their involvement with formal dementia-based support organizations (Jamieson et al., 2016; Kelsey et al., 2010). The following quote emphasizes the role these groups played to support families during transitions in care,

I’ve been going to an Alzheimer’s support group that’s sponsored through the state and the local association, and I think that’s extremely valuable for people . . . support groups are really helpful because they prepare you for the next step (Kelsey et al., 2010, p. 260).

#### Evaluating the quality of care associated with the health care transition

##### Questioning quality of care

Most of the papers reported experiences of poor care that stemmed from not considering personal needs, not delivering an adequate standard of care, and not disclosing patient harm (Bauer et al., 2011a, 2011b; Bloomer et al., 2016; Cloutier & Penning, 2017; Crawford et al., 2015; Digby et al., 2012; Fitzgerald et al., 2011; Henkusens et al., 2014; McLennon et al., 2019; Mockford et al., 2017; Parke et al., 2013).

Some caregivers described concerns about the quality of care being provided when they were not present (Crawford et al., 2015; Fitzgerald et al., 2011). In one paper by Bloomer et al. (2016), the authors included “Opportunities for improvement,” noting that caregivers identified concern over the treatment of patients with dementia: “I know the nurses have got to have enormous amount of patience. But the way that he was screamed at was terrible” (Bloomer et al., 2016, p. 1241). Caregiver narratives also indicated a level of tolerance toward perceived lack of competency on the part of clinical staff. They attributed this to limited experience, competing demands on a busy unit, insufficient time, declining education standards, and rigid regulations (Bauer et al., 2011a; Digby et al., 2012; Henkusens et al., 2014).

Bauer et al. (2011a) also reported that if health care professionals applied more personalized knowledge of the individual a better standard of care could have been provided, including an improved care transition. Similar sentiments were expressed by persons with dementia who experienced what is commonly referred to as “elderspeak” (i.e., referring to staff speaking to people with dementia in a condescending tone) (Digby et al., 2012).

Caregivers also played a key role at being vigilant of family members with dementia because of their concerns about poor quality care during health care transitions. Several authors reported caregivers being present and watchful citing concerns about safety and the desire to want to help (Bloomer et al., 2016; Digby et al., 2012; Jamieson et al., 2016). For example, one daughter from the study by Jamieson et al. (2016) expressed the following sentiment:

She was so confused; I ended up staying at the hospital the whole time. If I wasn’t there one of my sons would sit with her, she couldn’t understand where she was. I thought her safety was compromised, so it was safer for her, we felt, to have someone there with her (Jamieson et al., 2016, p. 1116).

##### Being satisfied with quality of care

Six studies included personal narratives that reflected positive feedback of the care provided and received. These instances tended to occur after a transition to long-term care (Bramble et al., 2009; Henkusens et al., 2014; Kelsey et al., 2010; Kiwi et al., 2018; McLennon et al., 2019), or for one study, to an assisted living setting (Hutchings et al., 2011). When care expectations were met by staff, “she is bathing and wearing clean clothes, her hair and grooming are being attended to, she is eating much better…” (Bramble et al., 2009, p. 3123), there was certainly a degree of satisfaction on the part of caregivers.

Connecting with either other residents or staff contributed to the perception of quality care. For example, Henkusens et al. (2014) paper about mealtime highlights the meaning of meals after experiencing a transition to long-term care. In this case, persons with dementia talked about “having company for supper” and building “camaraderie over supper,” and their caregivers speak about a “nice atmosphere.” The physical environment is also an important aspect of being satisfied with the care. For example, the presence of “flowers” (Hutchings et al., 2011), it being “bright…sunny” (Kelsey et al., 2010), and for Iranian participants, it is the presence of Persian food and language (Kiwi et al., 2018). In another study by Parke et al. (2013), the physical environment of the emergency department contributes to how caregivers and persons with dementia perceive the care experience. For example, the setting is largely perceived to be reliant on technology and task-oriented interactions. One caregiver describes the emergency as “The hustle and bustle in the cubicle area, the noise, the running around; it was like an uptight atmosphere and feeling…the buzzing around bothered Mom very badly” (Parke et al., 2013, p. 1213).

Four of the papers included reflections on care processes and the environment maintaining a sense of personal identify (Buse & Twigg, 2014; Cloutier & Penning, 2017; Digby et al., 2012; Henkusens et al., 2014). For example, Buse and Twigg (2014) examined how handbags supported identity and social roles during the transition to long-term care homes. Older women with dementia following the transition into residential care would keep their handbags and objects inside close to them, and their caregivers, likewise encouraged that behavior, “as a way of ‘keeping hold’ of an aspect of her” (Buse & Twigg, 2014, p. 7). Personal narratives of Cloutier and Penning (2017) actually revealed caregivers observance of the disintegration of their mothers’ identity in the following reflection,

…our personal narratives reflected how the broader care system (re)-identified our moms as problematic in terms of their behaviors (aggression and wandering) even though as family members, we knew our moms throughout their lives as shy and gentle women… (Cloutier & Penning, 2017, p. 78).

## Discussion

This review to our knowledge is the first to systematically search and synthesize the experiences and perceptions of persons with dementia and their caregivers undergoing health care transitions. Our analysis suggests three distinct yet overlapping categories rather than a linear process associated with the health care transition. Each category described several layered themes that demonstrated both the negative and positive experiences of participants; however, instances of dissatisfaction and discontent predominated the reported findings. While most narrative captured in the papers was from caregivers, some reflections from persons with dementia were included. We observed that persons with dementia and their caregivers shared similar experiences with regard to health care transitions, namely, wanting to be informed, looking forward to a new start post transition, and wanting to maintain their identity. However, caregivers seemed likely to notice fractured care transition processes and poor care, like rushed discharge planning, communication breakdowns, and substandard care practices, while persons with dementia spoke to establishing authentic and respectful connections with others and preserving their independence. Together these examples highlight potential areas of improvement in the transitional care of persons with dementia and their caregivers.

Synthesized data revealed an emotional response by both caregivers and persons with dementia to the care transition. During health care transitions, caregivers expressed feeling overwhelmed by complex care needs and frustrated by the invisibility of their usual role. Feelings of grief also emerged, and in contrast, acceptance of the transition was eventually experienced by some participants. This reflects findings of people with dementia being able to recognize their own physical and functional deterioration resulting in a move into care (Thein et al., 2011). Caregiver burden has been found to be predictive of institutionalization, although previous research suggests that only 15% of caregivers actually identify their burden as a reason for the transition (Etters et al., 2008; Gaugler et al. 2009). Health care transitions seem to trigger or exacerbate complicated grief reactions. The caregivers’ experience of guilt and relentless grief are consistent with literature on anticipatory grief defined as multiple losses for the caregiver (companionship, personal freedom, and control) and the person with dementia (Chan et al., 2013). Previous research reported the prevalence of anticipatory grief among caregivers between 47% and 71% (Chan et al., 2013; Holley & Mast, 2009). In our findings, the experience of feeling relief and adjusting to a new reality after a care transition were shown to be mutually experienced by caregivers and persons with dementia. Similar results were reported by Graneheim et al. (2014), when caregivers expressed relief when their family members received better care in a nursing home than what they could provide at home. Therefore, Blandin and Pepin’s (2017) work on the Dementia Grief Model aligns with our study demonstrating multifaceted, category of emotions, including feeling overwhelmed, frustrated, grief, and finally, acceptance.

Processes associated with the health care transition were often hindered by ineffective communication, poor care coordination and impromptu discharge planning. This review demonstrates inadequate information sharing between caregivers and health care providers at the point of transition resulting in caregivers being uninformed about the discharge plan and having to coordinate care post discharge. These findings are supported by previous research that suggests among patients with dementia leaving acute care, only 39.1% had a documented discharge plan, 30% had a named person coordinating their care, and less than half of caregivers (41.3%) had 24 h or more notice of the discharge (Timmons et al., 2016). The participant’s perception that they received insufficient information is also related to feeling disengaged from discharge planning processes. Supporting this finding is a systematic review that reported health care providers lack accountability and/or ability to effectively engage with family caregivers of older people with dementia resulting in caregivers being unaware of the patient’s discharge (Stockwell-Smith et al., 2018). Moreover, the health care transition experience had some caregivers relying on formal and informal networks of support for information and strongly advocating for better care for their loved one. This stands to support a growing body of literature on the concept of resilience in dementia caregiving (Conway et al. 2020; O’Dwyer et al., 2017; Teahan et al., 2018). The presence of resilience has been characterized as being proactive, determined, and resourceful on the part of caregivers (O’Dwyer et al., 2017). Supportive environmental factors included access to practical and emotional support (O’Dwyer et al., 2017). Previous research has shown that family caregivers are less likely to display negative psychological symptoms if they are satisfied with their social connections (Brodaty & Donkin, 2009).

Health care transitions also reveal how important quality of care is for people with dementia and their caregivers during care delivery. Other research has shown that 54% of caregivers expressed dissatisfaction with some aspect of care delivered in acute care of their cognitively impaired family member (Whittamore et al., 2014). Caregivers expressed varied experiences with the care provided, which heightened their vigilance and ongoing monitoring of the care. Our findings are also consistent with the work by Jurgens et al. (2012) that describes a “cycle of discontent.” Broadly, the cycle proposes that perceived unmet expectations by caregivers provoke uncertainty or suspiciousness, which leads to conflicted relationships with clinical staff (poor communication and tension over care). Our study identified additional insight that those living with dementia wanted not only more control over their care transition but also desired to maintain a sense of their individuality and connection with others. Similar findings of the importance of preserving culture, including food and traditions, were noted by immigrant caregivers’ of their non-English speaking relatives with dementia (Kong et al. 2010). In this case, transitional care offers an opportunity to focus on person-centered care, tailoring activities to the interest of the individual, identifying needs and providing individualized care (Heggestad et al., 2015; Smebye & Kirkevold, 2013).

### Strengths and limitations

This is the first meta-ethnography of the experiences and perceptions of older adults with dementia and their caregivers undertaking health care transitions. The systematic approach offered by a meta-ethnography allowed us to identify, analyze, and synthesize a range of qualitative studies on a prioritized area of health reform and research (Prusaczyk et al., 2020). In addition, the findings of the meta-ethnography highlighted several concepts and theories that support and relate to areas of priorities.

Although this review provides important insights into health care transition literature, it is not without limitations. The meta-ethnography may have missed other empirical studies having only one researcher screening the literature to determine eligibility. Although close to 1500 articles were searched by the first author, the presence of another independent reviewer might have helped to identify additional relevant studies. Similarly, this author initially conducted the analysis, however, to enhance rigor of the findings all authors, considered to be content experts, contributed to the analytical processes and decisions, and agreed with the final thematic results. The meta-ethnography approach itself, which relies on the interpretation and integration of researchers’ primary data, is inherently interpretive. Therefore, another group of researchers may have generated a different conceptual understanding. Although by outlining the first- and second-order constructs, some of the interpretation came directly from primary study authors and their participants. Last, authors did not conduct a negative case analysis, which may have led to identifying important differences (Morse, 2015).

### Implications for future research and practice

While we have been able to synthesize an important body of literature, several limitations have been identified. First, only three studies included direct quotes from individuals with dementia; rather, the voice of caregivers tended to be sought by authors. Future qualitative research would benefit from maximizing the inclusion of those with dementia by applying modified consent practices (Slaughter et al., 2007), using specific communication strategies (e.g., building rapport with participants and keeping questions general) (Digby et al., 2016; Murphy et al., 2015), and using other data collection methods that support interviews to overcome communication deficiencies (i.e., field notes) (Phillipson & Hammond, 2018). Practice and policy reforms in health care systems are more impactful when they are informed by an understanding of persons with dementia (Novek & Wilkinson, 2019).

The studies took place across a range of countries, each with different health care systems and organizational contexts. Understanding the connections between setting characteristics and care transition experiences will be important in designing future care transition practices to support people with dementia and their caregivers. To that end, it was not clear in the included studies the extent to which system-level challenges, unique to different health systems and organizational contexts, impacted the health care transition of the persons with dementia and their families. For example, participant reports of experiencing pressurized discharge practices may reflect misalignment between the organizational level goals and pressures outweighing the need to improve micro level care practices (Conn et al., 2018). Future research can explore and develop models of transitional care that help to mitigate system-level deficiencies and improve the health care transitions of patients and caregivers. To that end, we can gain a better understanding of “dementia-friendly” contexts to support better care transitions.

Several practice implications are also apparent from the findings of this review. We noted that caregiver needs assessment were not routinely mentioned to have been conducted. Previous research has shown that post transition, families feel unprepared to take on the caregiver role and feel that staff neglected the component of discharge planning relating to accessing assistance and resources in the community (Grimmer et al., 2004; Timmons et al., 2016). Therefore, it is imperative that health care providers are equipped to provide relationship-centered care by involving both impacted person and caregivers in assessing treatment preferences, transition preferences, and caregiver support needs in advance, during, and after the transition (Nolan et al., 2004). In doing so, these screening and assessment tools may help to enhance caregiver resilience. Given the fact that caregivers across studies seemed to experience a complex grief response represented by the Dementia Grief Model suggests that families and persons with dementia may benefit from more psychological support. With that said, there is a call to action to develop more robust evidence-informed best practices in care transitions of dementia patients and their caregivers. Our meta-ethnography has elucidated important factors to both the effected person and caregivers when experiencing a health care transition—feeling informed, feeling connecting, and having a say/choice.

### Conclusion

A systematic search and review of the qualitative literature revealed a layered understanding of the care transitions experiences and perceptions of some people living with dementia and mostly their caregivers. Findings show an interconnected experience of feelings, processes, and evaluation of the quality of care related to the health care transition. This meta-ethnography has also demonstrated that persons with dementia have been minimally included in qualitative research on health care transitions. Even among studies that included persons with dementia, few of their quotes were used. We strongly recommend further qualitative research that promotes their involvement so that transitional care interventions can be better adapted to the needs of people living with dementia.

## Supplemental Material

sj-pdf-1-dem-10.1177_14713012211031779 – Supplemental Material for Using meta-ethnography to understand the care transition experience of people with dementia and their caregivers Click here for additional data file. Supplemental Material, sj-pdf-1-dem-10.1177_14713012211031779 for Using meta-ethnography to understand the care transition experience of people with dementia and their caregivers by Marianne Saragosa, Lianne Jeffs, Karen Okrainec and Kerry Kuluski in Dementia

## References

[bibr1-14713012211031779] Alzheimer’s Association . (2019). 2019 Alzheimer’s disease facts and figures. Alzheimer’s & dementia, 15(3), 321-387.

[bibr2-14713012211031779] Arora V. M. Prochaska M. L. Farnan J. M. D’Arcy V. M. J. Schwanz K. J. Vinci L. M. Davis A. M. Meltzer D. O. Johnson J. K. (2010). Problems after discharge and understanding of communication with their primary care physicians among hospitalized seniors: A mixed methods study. Journal of Hospital Medicine, 5(7), 385-391. 20578045 10.1002/jhm.668 PMC3186075

[bibr3-14713012211031779] Atkins S. Lewin S. Smith H. Engel M. Fretheim A. Volmink J. (2008). Conducting a meta-ethnography of qualitative literature: Lessons learnt. BMC Medical Research Methodology, 8(1), 21. 18416812 10.1186/1471-2288-8-21 PMC2374791

[bibr4-14713012211031779] Bauer M. Fitzgerald L. Koch S. (2011a). Hospital discharge as experienced by family carers of people with dementia: A case for quality improvement. Journal for Healthcare Quality, 33(6), 9-16. 10.1111/j.1945-1474.2011.00122.x 22103700

[bibr5-14713012211031779] Bauer M. Fitzgerald L. Koch S. King S. (2011b). How family carers view hospital discharge planning for the older person with a dementia. Dementia, 10(3), 317-323.

[bibr6-14713012211031779] Bethell J. Commisso E. Rostad H. M. Puts M. Babineau J. Grinbergs-Saull A. Wighton M. B. Hammel J. Doyle E. Nadeau S. McGilton K. S. (2018). Patient engagement in research related to dementia: A scoping review. Dementia, 17(8), 944-975. 30373460 10.1177/1471301218789292

[bibr7-14713012211031779] Blandin K. Pepin R. (2017). Dementia grief: A theoretical model of a unique grief experience. Dementia, 16(1), 67-78. 25883036 10.1177/1471301215581081 PMC4853283

[bibr8-14713012211031779] Bloomer M. Digby R. Tan H. Crawford K. Williams A. (2016). The experience of family carers of people with dementia who are hospitalised. Dementia, 15(5), 1234-1245. 25394556 10.1177/1471301214558308

[bibr9-14713012211031779] Bramble M. Moyle W. McAllister M. (2009). Seeking connection: Family care experiences following long-term dementia care placement. Journal of Clinical Nursing, 18(22), 3118-3125. 19825115 10.1111/j.1365-2702.2009.02878.x

[bibr10-14713012211031779] Britten N. Campbell R. Pope C. Donovan J. Morgan M. Pill R. (2002). Using meta ethnography to synthesise qualitative research: A worked example. Journal of Health Services Research & Policy, 7(4), 209-215. 12425780 10.1258/135581902320432732

[bibr11-14713012211031779] Brodaty H. Donkin M. (2009). Family caregivers of people with dementia. Dialogues in Clinical Neuroscience, 11(2), 217-228. 19585957 10.31887/DCNS.2009.11.2/hbrodaty PMC3181916

[bibr12-14713012211031779] Buse C. Twigg J. (2014). Women with dementia and their handbags: Negotiating identity, privacy and ‘home’ through material culture. Journal of Aging Studies, 30, 14-22. 24984904 10.1016/j.jaging.2014.03.002

[bibr13-14713012211031779] Callahan K. E. Lovato J. F. Miller M. E. Easterling D. Snitz B. Williamson J. D. (2015a). Associations between mild cognitive impairment and hospitalization and readmission. Journal of the American Geriatrics Society, 63(9), 1880-1885. 26313420 10.1111/jgs.13593 PMC4809245

[bibr14-14713012211031779] Callahan C. M. Tu W. Unroe K. T. LaMantia M. A. Stump T. E. Clark D. O. (2015b). Transitions in care in a nationally representative sample of older Americans with dementia. Journal of the American Geriatrics Society, 63(8), 1495-1502. 26200764 10.1111/jgs.13540 PMC4834213

[bibr15-14713012211031779] Chan D. Livingston G. Jones L. Sampson E. L. (2013). Grief reactions in dementia carers: A systematic review. International Journal of Geriatric Psychiatry, 28(1), 1-17. 22407743 10.1002/gps.3795

[bibr16-14713012211031779] Charmaz K. (2014). Constructing grounded theory. Sage.

[bibr17-14713012211031779] Cheek J. Ballantyne A. Gillham D. Mussared J. Flett P. Lewin G. Walker M. Roder‐Allen G. Quan J. Vandermeulen S. (2006). Improving care transitions of older people: Challenges for today and tomorrow. Quality in Ageing and Older Adults, 7(4), 18-26.

[bibr18-14713012211031779] Chenoweth L. Kable A. Pond D. (2015). Research in hospital discharge procedures addresses gaps in care continuity in the community, but leaves gaping holes for people with dementia: A review of the literature. Australasian Journal on Ageing, 34(1), 9-14. 10.1111/ajag.12205 25735471

[bibr19-14713012211031779] Cloutier D. S. Penning M. J. (2017). Janus at the crossroads: Perspectives on long-term care trajectories for older women with dementia in a Canadian context. The Gerontologist, 57(1), 68-81. 27852640 10.1093/geront/gnw158 PMC5241789

[bibr20-14713012211031779] Coleman E. A. (2003). Falling through the cracks: Challenges and opportunities for improving transitional care for persons with continuous complex care needs. Journal of the American Geriatrics Society, 51(4), 549-555. 12657078 10.1046/j.1532-5415.2003.51185.x

[bibr21-14713012211031779] Comans T. A. Peel N. M. Hubbard R. E. Mulligan A. D. Gray L. C. Scuffham P. A. (2016). The increase in healthcare costs associated with frailty in older people discharged to a post-acute transition care program. Age and Ageing, 45(2), 317-320. 26769469 10.1093/ageing/afv196

[bibr22-14713012211031779] Conn L. G. Zwaiman A. DasGupta T. Hales B. Watamaniuk A. Nathens A. B. (2018). Trauma patient discharge and care transition experiences: Identifying opportunities for quality improvement in trauma centres. Injury, 49(1), 97-103. 28988066 10.1016/j.injury.2017.09.028

[bibr23-14713012211031779] Conway L. Wolverson E. Clarke C. (2020). Shared experiences of resilience amongst couples where one partner is living with dementia-A grounded theory study. Frontiers in Medicine, 7, 219. 32582728 10.3389/fmed.2020.00219 PMC7296104

[bibr24-14713012211031779] Crawford K. Digby R. Bloomer M. Tan H. Williams A. (2015). Transitioning from caregiver to visitor in a long-term care facility: The experience of caregivers of people with dementia. Aging & Mental Health, 19(8), 739-746. 25266371 10.1080/13607863.2014.962008

[bibr25-14713012211031779] Digby R. Lee S. Williams A. (2016). Interviewing people with dementia in hospital: Recommendations for researchers. Journal of Clinical Nursing, 25(7-8), 1156-1165. 26879137 10.1111/jocn.13141

[bibr26-14713012211031779] Digby R. Moss C. Bloomer M. (2012). Transferring from an acute hospital and settling into a subacute facility: The experience of patients with dementia. International Journal of Older People Nursing, 7(1), 57-64. 21707932 10.1111/j.1748-3743.2011.00282.x

[bibr27-14713012211031779] Dixon-Woods M. Sutton A. Shaw R. Miller T. Smith J. Young B. Bonas S. Booth A. Jones D. R. (2007). Appraising qualitative research for inclusion in systematic reviews: A quantitative and qualitative comparison of three methods. Journal of Health Services Research & Policy, 12(1), 42-47. 17244397 10.1258/135581907779497486

[bibr28-14713012211031779] Doyle L. H. (2003). Synthesis through meta-ethnography: Paradoxes, enhancements, and possibilities. Qualitative Research, 3(3), 321-344.

[bibr29-14713012211031779] Etters L. Goodall D. Harrison B. E. (2008). Caregiver burden among dementia patient caregivers: A review of the literature. Journal of the American Academy of Nurse Practitioners, 20(8), 423-428. 18786017 10.1111/j.1745-7599.2008.00342.x

[bibr30-14713012211031779] Finfgeld D. L. (2003). Metasynthesis: The state of the art-so far. Qualitative Health Research, 13(7), 893-904. 14502956 10.1177/1049732303253462

[bibr31-14713012211031779] Fitzgerald L. R. Bauer M. Koch S. H. King S. J. (2011). Hospital discharge: Recommendations for performance improvement for family carers of people with dementia. Australian Health Review, 35(3), 364-370. 21871200 10.1071/AH09811

[bibr32-14713012211031779] France E. F. Cunningham M. Ring N. Uny I. Duncan E. A. S. Jepson R. G. Maxwell M. Roberts R. J. Turley R. L. Booth A. Britten N. Flemming K. Gallagher I. Garside R. Hannes K. Lewin S. Noblit G. W. Pope C. Thomas J. Noyes J. (2019). Improving reporting of meta-ethnography: the eMERGe reporting guidance. BMC Medical Research Methodology, 19(1), 25. 30709371 10.1186/s12874-018-0600-0 PMC6359764

[bibr33-14713012211031779] Gaugler J. E. Yu F. Krichbaum K. Wyman J. F. (2009). Predictors of nursing home admission for persons with dementia. Medical care, 47, 191-198. 19169120 10.1097/MLR.0b013e31818457ce

[bibr34-14713012211031779] Goldberg A. Straus S. E. Hamid J. S. Wong C. L. (2015). Room transfers and the risk of delirium incidence amongst hospitalized elderly medical patients: A case-control study. BMC Geriatrics, 15(1), 69. 26108254 10.1186/s12877-015-0070-8 PMC4478641

[bibr35-14713012211031779] Graneheim U. H. Johansson A. Lindgren B.-M. (2014). Family caregivers’ experiences of relinquishing the care of a person with dementia to a nursing home: Insights from a meta-ethnographic study. Scandinavian Journal of Caring Sciences, 28(2), 215-224. 23578033 10.1111/scs.12046

[bibr36-14713012211031779] Grimmer K. Moss J. Falco J. (2004). Becoming a carer for an elderly person after discharge from an acute hospital admission. Internet Journal of Allied Health Sciences and Practice, 2(4), 4.

[bibr37-14713012211031779] Gruneir A. Bronskill S. Bell C. Gill S. Schull M. Ma X. Anderson G. Rochon P. A. (2012). Recent health care transitions and emergency department use by chronic long term care residents: A population-based cohort study. Journal of the American Medical Directors Association, 13(3), 202-206. 22056922 10.1016/j.jamda.2011.10.001

[bibr38-14713012211031779] Heggestad A. K. T. Nortvedt P. Slettebø Å. (2015). Dignity and care for people with dementia living in nursing homes. Dementia, 14(6), 825-841. 24381212 10.1177/1471301213512840

[bibr39-14713012211031779] Henkusens C. Keller H. H. Dupuis S. Schindel Martin L. (2014). Transitions to long-term care. Journal of Applied Gerontology, 33(5), 541-563. 24652920 10.1177/0733464813515091

[bibr40-14713012211031779] Hirschman K. B. Hodgson N. A. (2018). Evidence-based interventions for transitions in care for individuals living with dementia. The Gerontologist, 58(Suppl 1), S129-S140. 29361067 10.1093/geront/gnx152

[bibr41-14713012211031779] Holley C. K. Mast B. T. (2009). The impact of anticipatory grief on caregiver burden in dementia caregivers. The Gerontologist, 49(3), 388-396. 19386826 10.1093/geront/gnp061

[bibr42-14713012211031779] Hutchings D. Wells J. J. L. O’Brien K. Wells C. Alteen A. M. Cake L. J. (2011). From institution to ‘home’: Family perspectives on a unique relocation process. Canadian Journal on Aging/La Revue canadienne du vieillissement, 30(2), 223-232. 10.1017/S0714980811000043 24650671

[bibr43-14713012211031779] Jamieson M. Grealish L. Brown J.-A. Draper B. (2016). Carers: The navigators of the maze of care for people with dementia-A qualitative study. Dementia, 15(5), 1112-1123. 25305279 10.1177/1471301214554930

[bibr44-14713012211031779] Jurgens F. J. Clissett P. Gladman J. R. Harwood R. H. (2012). Why are family carers of people with dementia dissatisfied with general hospital care? A qualitative study. BMC Geriatrics, 12(1), 57. 23006826 10.1186/1471-2318-12-57 PMC3509004

[bibr45-14713012211031779] Kelsey S. G. Laditka S. B. Laditka J. N. (2010). Caregiver perspectives on transitions to assisted living and memory care. American Journal of Alzheimer’s Disease and Other Dementias, 25(3), 255-264. 10.1177/1533317509357737 PMC10845508 20147600

[bibr46-14713012211031779] Kiwi M. Hydén L.-C. Antelius E. (2018). Deciding upon transition to residential care for persons living with dementia: Why do Iranian family caregivers living in Sweden cease caregiving at home? Journal of Cross-Cultural Gerontology, 33(1), 21-42. 29170865 10.1007/s10823-017-9337-1 PMC5845599

[bibr47-14713012211031779] Kong E.-H. Deatrick J. A. Evans L. K. (2010). The experiences of Korean immigrant caregivers of non-English-speaking older relatives with dementia in American nursing homes. Qualitative Health Research, 20(3), 319-329. 19940089 10.1177/1049732309354279

[bibr48-14713012211031779] McLennon S. M. Davis A. Covington S. Anderson J. G. (2019). “At the end we feel forgotten”: Needs, concerns, and advice from blogs of dementia family caregivers. Clinical Nursing Research, 30(1), 82-88. 31387367 10.1177/1054773819865871

[bibr49-14713012211031779] Mockford C. Seers K. Murray M. Oyebode J. Clarke R. Staniszewska S. Suleman R. Boex S. Diment Y. Grant R. Leach J. Sharma U. (2017). The development of service user-led recommendations for health and social care services on leaving hospital with memory loss or dementia - The SHARED study. Health Expectations, 20(3), 495-507. 27389613 10.1111/hex.12477 PMC5433530

[bibr50-14713012211031779] Mondor L. Maxwell C. J. Hogan D. B. Bronskill S. E. Gruneir A. Lane N. E. Wodchis W. P. (2017). Multimorbidity and healthcare utilization among home care clients with dementia in Ontario, Canada: A retrospective analysis of a population-based cohort. PLoS medicine, 14(3), e1002249. 28267802 10.1371/journal.pmed.1002249 PMC5340355

[bibr51-14713012211031779] Morse J. M. (2015). Critical analysis of strategies for determining rigor in qualitative inquiry. Qualitative Health Research, 25(9), 1212-1222. 26184336 10.1177/1049732315588501

[bibr52-14713012211031779] Mudge A. M. Shakhovskoy R. Karrasch A. (2013). Quality of transitions in older medical patients with frequent readmissions: Opportunities for improvement. European Journal of Internal Medicine, 24(8), 779-783. 24055382 10.1016/j.ejim.2013.08.708

[bibr53-14713012211031779] Mueller C. Lautenschlaeger S. Meyer G. Stephan A. (2017). Interventions to support people with dementia and their caregivers during the transition from home care to nursing home care: A systematic review. International Journal of Nursing Studies, 71, 139-152. 28411508 10.1016/j.ijnurstu.2017.03.013

[bibr54-14713012211031779] Murphy K. Jordan F. Hunter A. Cooney A. Casey D. (2015). Articulating the strategies for maximising the inclusion of people with dementia in qualitative research studies. Dementia, 14(6), 800-824. 24403314 10.1177/1471301213512489

[bibr55-14713012211031779] Naylor M. D. Aiken L. H. Kurtzman E. T. Olds D. M. Hirschman K. B. (2011). The importance of transitional care in achieving health reform. Health affairs, 30(4), 746-754. 21471497 10.1377/hlthaff.2011.0041

[bibr56-14713012211031779] Noblit G. W. Hare R. D. (1988). Meta-ethnography: Synthesizing qualitative studies (Vol. 11). Sage.

[bibr57-14713012211031779] Nolan M. R. Davies S. Brown J. Keady J. Nolan J. (2004). Beyond ‘person-centred’ care: A new vision for gerontological nursing. Journal of Clinical Nursing, 13, 45-53. 15028039 10.1111/j.1365-2702.2004.00926.x

[bibr58-14713012211031779] Novek S. Wilkinson H. (2019). Safe and inclusive research practices for qualitative research involving people with dementia: A review of key issues and strategies. Dementia, 18(3), 1042-1059. 28350179 10.1177/1471301217701274

[bibr59-14713012211031779] O’Dwyer S. T. Moyle W. Taylor T. Creese J. Zimmer-Gembeck M. (2017). In their own words: How family carers of people with dementia understand resilience. Behavioral Sciences, 7(3), 57. 10.3390/bs7030057 PMC5618065 28825686

[bibr60-14713012211031779] Parke B. Hunter K. F. Strain L. A. Marck P. B. Waugh E. H. McClelland A. J. (2013). Facilitators and barriers to safe emergency department transitions for community dwelling older people with dementia and their caregivers: A social ecological study. International Journal of Nursing Studies, 50(9), 1206-1218. 23219329 10.1016/j.ijnurstu.2012.11.005

[bibr61-14713012211031779] Phillipson L. Hammond A. (2018). More than talking: A scoping review of innovative approaches to qualitative research involving people with dementia. International Journal of Qualitative Methods, 17(1), 1609406918782784.

[bibr62-14713012211031779] Piraino E. Heckman G. Glenny C. Stolee P. (2012). Transitional care programs: Who is left behind? A systematic review. International Journal of Integrated Care, 12, e132. 23593046 10.5334/ijic.805 PMC3601531

[bibr63-14713012211031779] Prince M. Bryce R. Albanese E. Wimo A. Ribeiro W. Ferri C. P. (2013). The global prevalence of dementia: A systematic review and metaanalysis. Alzheimer’s & Dementia, 9(1), 63-75. 10.1016/j.jalz.2012.11.007 23305823

[bibr64-14713012211031779] Prusaczyk B. Fabbre V. Morrow-Howell N. Proctor E. (2020). Understanding transitional care provided to older adults with and without dementia: A mixed methods study. International Journal of Care Coordination, 23(1), 14-23.

[bibr65-14713012211031779] Public Health Agency of Canada . (2014). Mapping connections: An understanding of neurological conditions in Canada. Public Health Agency of Canada.

[bibr66-14713012211031779] Public Health Agency of Canada . (2019). A dementia strategy for Canada: Together we aspire. Retrieved from https://www.canada.ca/en/public-health/services/publications/diseases-conditions/dementia-strategy.html

[bibr67-14713012211031779] Purc‐Stephenson R. J. Thrasher C. (2010). Nurses’ experiences with telephone triage and advice: A meta‐ethnography. Journal of advanced nursing, 66(3), 482-494. 20423383 10.1111/j.1365-2648.2010.05275.x

[bibr68-14713012211031779] Scottish Government . (2017). National dementia strategy: 2017-2020. Retrieved from https://www.gov.scot/publications/scotlands-national-dementia-strategy-2017-2020/.

[bibr69-14713012211031779] Slaughter S. Cole D. Jennings E. Reimer M. A. (2007). Consent and assent to participate in research from people with dementia. Nursing Ethics, 14(1), 27-40. 17334168 10.1177/0969733007071355

[bibr70-14713012211031779] Smebye K. L. Kirkevold M. (2013). The influence of relationships on personhood in dementia care: A qualitative, hermeneutic study. BMC Nursing, 12(1), 29. 24359589 10.1186/1472-6955-12-29 PMC3878215

[bibr71-14713012211031779] Stockwell‐Smith G. Moyle W. Marshall A. P. Argo A. Brown L. Howe S. Layton K. Naidoo O. Santoso Y. Soleil-Moudiky-Joh E. Grealish L. (2018). Hospital discharge processes involving older adults living with dementia: An integrated literature review. Journal of Clinical Nursing, 27(5-6), e712-e725. 29076202 10.1111/jocn.14144

[bibr72-14713012211031779] Sury L. Burns K. Brodaty H. (2013). Moving in: Adjustment of people living with dementia going into a nursing home and their families. International Psychogeriatrics, 25(6), 867-876. 23425369 10.1017/S1041610213000057

[bibr73-14713012211031779] Taylor J. S. DeMers S. M. Vig E. K. Borson S. (2012). The disappearing subject: Exclusion of people with cognitive impairment and dementia from geriatrics research. Journal of the American Geriatrics Society, 60(3), 413-419. 22288835 10.1111/j.1532-5415.2011.03847.x

[bibr74-14713012211031779] Teahan Á. Lafferty A. McAuliffe E. Phelan A. O'Sullivan L. O’Shea D. Fealy G. (2018). Resilience in family caregiving for people with dementia: A systematic review. International Journal of Geriatric Psychiatry, 33(12), 1582-1595. 30230018 10.1002/gps.4972

[bibr75-14713012211031779] Teno J. M. Gozalo P. Trivedi A. N. Bunker J. Lima J. Ogarek J. Mor V. (2018). Site of death, place of care, and health care transitions among US Medicare beneficiaries, 2000-2015. JAMA, 320(3), 264-271. 29946682 10.1001/jama.2018.8981 PMC6076888

[bibr76-14713012211031779] Thein N. W. D’Souza G. Sheehan B. (2011). Expectations and experience of moving to a care home: Perceptions of older people with dementia. Dementia, 10(1), 7-18.

[bibr77-14713012211031779] Timmons S. O’Shea E. O’Neill D. Gallagher P. de Siún A. McArdle D. Gibbons P. Kennelly S. (2016). Acute hospital dementia care: Results from a national audit. BMC Geriatrics, 16(1), 113. 27245979 10.1186/s12877-016-0293-3 PMC4886443

[bibr78-14713012211031779] Tong A. Sainsbury P. Craig J. (2007). Consolidated criteria for reporting qualitative research (COREQ): A 32-item checklist for interviews and focus groups. International Journal for Quality in Health Care, 19(6), 349-357. 17872937 10.1093/intqhc/mzm042

[bibr79-14713012211031779] Whittamore K. H. Goldberg S. E. Bradshaw L. E. Harwood R. H., & Medical Crises in Older Pepole Study Group, (2014). Factors associated with family caregiver dissatisfaction with acute hospital care of older cognitively impaired relatives. Journal of the American Geriatrics Society, 62(12), 2252-2260. 25516022 10.1111/jgs.13147

[bibr80-14713012211031779] Wladkowski S. P. (2016). Live discharge from hospice and the grief experience of dementia caregivers. Journal of Social Work in End-of-Life & Palliative Care, 12(1-2), 47-62. 27143573 10.1080/15524256.2016.1156600

[bibr81-14713012211031779] World Health Organization . (2016). Transitions of care: Technical series on safer primary care. Retrieved from https://apps.who.int/iris/bitstream/handle/10665/252272/9789241511599-eng.pdf;jsessionid=0A64676449604CA0FE807DC9F310BF1E?sequence=1

[bibr82-14713012211031779] World Health Organization . (2019). Dementia key facts. Retrieved from https://www.who.int/news-room/fact-sheets/detail/dementia

