# Supplementary material for: Using meta-ethnography to understand the care transition experience of people with dementia and their caregivers
Source: Dementia (London). 2021 Aug 2;21(1):153–80. doi: 10.1177/14713012211031779 (PMC8721620; doi:10.1177/14713012211031779)
Supplement: sj-pdf-1-dem-10.1177_14713012211031779 – Supplemental Material for Using meta-ethnography to understand the care transition experience of people with dementia and their caregivers [file sj-pdf-1-dem-10.1177_14713012211031779.pdf]

MEDLINE Hospital to Home Meta Ethno

1. exp QUALITATIVE RESEARCH/ or qualitative.mp.
2. "focus group".mp. or exp Focus Groups/
3. "participant observation".mp.
4. "nonparticipant observation".mp.
5. ("perspectiv*" or "“Perce*” OR “view*” OR “Belie*” OR “experience* OR “attitude*” OR “opinion*” OR “feel*” OR “Understand*” OR “Subjective” OR “thought*").mp. [mp=title, abstract, original title, name of substance word, subject heading word, floating sub-heading word, keyword heading word, organism supplementary concept word, protocol supplementary concept word, rare disease supplementary concept word, unique

identifier, synonyms]

6. (qualitative adj3 research).mp. [mp=title, abstract, original title, name of substance word, subject heading word, floating sub-heading word, keyword heading word, organism supplementary concept word, protocol supplementary concept word, rare disease supplementary concept word, unique identifier, synonyms]

7. (interview adj1 guide).mp. [mp=title, abstract, original title, name of substance word, subject heading word, floating sub-heading word, keyword heading word, organism supplementary concept word, protocol supplementary concept word, rare disease

supplementary
concept word, unique
identifier, synonyms]

8. phenomenol*.mp.

9. ethnograph*.mp.
[mp=title, abstract,
original title, name of
substance word,
subject heading word,
floating sub-heading
word, keyword
heading word,
organism
supplementary
concept word,
protocol
supplementary
concept word, rare
disease
supplementary
concept word, unique
identifier, synonyms]

10. (grounded adj1
theory).mp. [mp=title,
abstract, original title,
name of substance
word, subject heading
word, floating sub-
heading word,
keyword heading
word, organism
supplementary
concept word,
protocol
supplementary

concept word, rare disease supplementary concept word, unique identifier, synonyms]

11. "grounded approach".mp. [mp=title, abstract, original title, name of substance word, subject heading word, floating sub-heading word, keyword heading word, organism supplementary concept word, protocol supplementary concept word, rare disease supplementary concept word, unique identifier, synonyms]

12. 1 or 2 or 3 or 4 or 5 or 6 or 7 or 8 or 9 or 10 or 11

13. "life experience".mp. [mp=title, abstract, original title, name of substance word, subject heading word, floating sub-heading word, keyword

heading word,
organism
supplementary
concept word,
protocol
supplementary
concept word, rare
disease
supplementary
concept word, unique
identifier, synonyms]

14. 12 or 13

15. exp DEMENTIA/
or exp
FRONTOTEMPORAL
DEMENTIA/ or exp
DEMENTIA,
VASCULAR/ or exp
DEMENTIA, MULTI-
INFARCT/

16. exp DELIRIUM/
or delirium.mp.

17. (cognitive adj3
disorders).mp.
[mp=title, abstract,
original title, name of
substance word,
subject heading word,
floating sub-heading
word, keyword
heading word,
organism
supplementary
concept word,
protocol

supplementary
concept word, rare
disease
supplementary
concept word, unique
identifier, synonyms]

18. (senile adj1
dementia).mp.
[mp=title, abstract,
original title, name of
substance word,
subject heading word,
floating sub-heading
word, keyword
heading word,
organism
supplementary
concept word,
protocol
supplementary
concept word, rare
disease
supplementary
concept word, unique
identifier, synonyms]

19. (cogniti* adj1
impair*).mp.
[mp=title, abstract,
original title, name of
substance word,
subject heading word,
floating sub-heading
word, keyword
heading word,
organism

supplementary
concept word,
protocol
supplementary
concept word, rare
disease
supplementary
concept word, unique
identifier, synonyms]

20. alzheimer's disease.mp. or exp Alzheimer Disease/

21. "memory loss".mp. [mp=title, abstract, original title, name of substance word, subject heading word, floating sub-heading word, keyword heading word, organism supplementary concept word, protocol supplementary concept word, rare disease supplementary concept word, unique identifier, synonyms]

22. (dementia adj1 care).mp. [mp=title, abstract, original title, name of substance word, subject heading

word, floating sub-heading word, keyword heading word, organism supplementary concept word, protocol supplementary concept word, rare disease supplementary concept word, unique identifier, synonyms]

23. 15 or 16 or 17 or 18 or 19 or 20 or 21 or 22

24. transitional care.mp. or exp "Continuity of Patient Care"/ or exp Transitional Care/ or exp Patient Discharge/

25. patient transfer.mp. or exp Patient Transfer/

26. discharge planning.mp.

27. (care adj1 transition).mp. [mp=title, abstract, original title, name of substance word, subject heading word, floating sub-heading

word, keyword
heading word,
organism
supplementary
concept word,
protocol
supplementary
concept word, rare
disease
supplementary
concept word, unique
identifier, synonyms]

28. care
transition.mp.

29. "transitions in
care".mp. [mp=title,
abstract, original title,
name of substance
word, subject heading
word, floating sub-
heading word,
keyword heading
word, organism
supplementary
concept word,
protocol
supplementary
concept word, rare
disease
supplementary
concept word, unique
identifier, synonyms]

30. (care adj3
transition).mp.
[mp=title, abstract,

original title, name of substance word, subject heading word, floating sub-heading word, keyword heading word, organism supplementary concept word, protocol supplementary concept word, rare disease supplementary concept word, unique identifier, synonyms]

31. (patient adj3 (discharge or transfer)).mp. [mp=title, abstract, original title, name of substance word, subject heading word, floating sub-heading word, keyword heading word, organism supplementary concept word, protocol supplementary concept word, rare disease supplementary concept word, unique

identifier, synonyms]

32. (transition* adj3 care).mp. [mp=title, abstract, original title, name of substance word, subject heading word, floating sub-heading word, keyword heading word, organism supplementary concept word, protocol supplementary concept word, rare disease supplementary concept word, unique identifier, synonyms]

33. case management.mp. or exp Case Management/

34. 24 or 25 or 26 or 27 or 28 or 29 or 30 or 31 or 32 or 33

35. 14 and 23 and 34

36. limit 35 to english language

37. limit 36 to yr="2009 - 2019"
